# Pathways of Worry During the Transition to Adolescence: An Exploration of Students’ Emotion Regulation, Metacognitive Beliefs and Coping

**DOI:** 10.3390/jintelligence13080090

**Published:** 2025-07-22

**Authors:** Yiran Ge, Andrew Kenneth Tolmie

**Affiliations:** 1 Department of Experimental Psychology, University of Oxford, Oxford OX1 2JD, UK; yiran.ge@psy.ox.ac.uk; 2 Department of Psychology and Human Development, Institute of Education, University College London, 20 Bedford Way, London WC1H 0AL, UK

**Keywords:** emotion regulation, emotional awareness, metacognitive beliefs, cognitive self-consciousness, emotion controllability belief, coping, adolescence, worry

## Abstract

This study examined how two metacognitive constructs, cognitive self-consciousness and beliefs about emotion regulation, mediate the link among early adolescents between emotion regulation and engagement in coping with worry, and whether these relationships change with age during this period. A total of 338 Chinese pupils completed a series of measures assessing the metacognitive constructs plus emotional awareness and regulation; scenario-based questions examined coping strategies. Participants were divided into two age groups, 11 to 12 (*M_age_* = 11.9 years) and 13 to 15 (*M_age_* = 13.2 years). Path models showed that younger participants adopted emotion-focused coping whereas older participants adopted more problem-focused coping, and these response patterns were mediated as hypothesized by cognitive self-consciousness and controllability beliefs towards worry. These findings highlight the need for more adaptive coping to be specifically targeted during early adolescence by raising awareness of controllability beliefs.

## 1. Introduction

During the transition from childhood to adolescence, emotional changes may contribute to the onset and maintenance of students’ stress and worrying (Esbjorn et al. 2015; Hankin 2015; Mathews et al. 2014). Common stress-related mental health issues, such as anxiety and depression, may be continually exacerbated by growing worries (Anniko et al. 2019). However, the impact of worry varies based on individuals’ cognitive–affective capabilities, such as monitoring negative thinking and implementing emotion regulation (ER) and coping strategies (Compas et al. 2017). Emotion regulation enables the modulation of personal reactions to emotional experiences while coping ranges from managing feelings to tackling external situations that provoke reactions, i.e., emotion-focused, problem-focused, and social (support seeking) coping (Carver et al. 1989; Eastabrook et al. 2014; Lazarus and Folkman 1984; Wang and Saudino 2011). Little research has examined the cognitive mechanisms underlying the use of these coping strategies, despite these being crucial in adjustment (Leipold et al. 2019; Min et al. 2013; O’Dowd et al. 2018; Troy and Mauss 2011).

Three cognitive factors may guide appropriate emotion regulation and coping responses: emotional awareness, controllability beliefs toward emotions, and cognitive self-consciousness. Emotional awareness involves the perception as well as identification of emotional states, enabling further actions to deal with them (Eastabrook et al. 2014), and has therefore been argued to constitute an early stage of emotion regulation (Gross 2015). Controllability beliefs are beliefs in whether emotions can be changed or controlled, which are associated with the use of different emotion regulation strategies and coping, depending on the level of perceived controllability (Ford and Gross 2019; Tamir et al. 2007). Cognitive self-consciousness is defined as individual reflection on the source of internal states in line with emotion beliefs, potentially facilitating the modification of emotion-provoking situations (Mansueto et al. 2022; Niedenthal et al. 2006; Wells and Cartwright-Hatton 2004). These latter two metacognitive constructs emerge at a later stage of emotional experience, referring to the internal process involving the evaluation and regulation of affect, than emotional awareness, as both entail making judgements about identified emotions (Georghiades 2004). Since both emotional awareness and metacognitive thinking develop rapidly in early adolescence (Buckley and Saarni 2006; Eastabrook et al. 2014), there are likely to be age variations in the link between these constructs and coping. Despite this, there have been limited studies sampling early adolescents. The present research therefore aimed to model the role of emotional awareness and metacognitive constructs in how early adolescents respond to worry, with a focus on age-related variation (Palmier-Claus et al. 2011).

### 1.1. Emotion Regulation and Coping

Emotion regulation (ER) involves adjusting either negative or positive emotion responses within oneself. ER has been described as the set of “extrinsic and intrinsic processes responsible for monitoring, evaluating, and modifying emotional reactions” (Thompson 1994, pp. 27–28) that essentially pertain to internal responses (Cole et al. 2004; Compas et al. 2014). In contrast, coping has been defined as the “cognitive and behavioral efforts to manage specific external and/or internal demands that are appraised as taxing or exceeding the resources of the person” and is restricted to tackling negative reactions (Lazarus and Folkman 1984, p. 141; Wang and Saudino 2011). Coping comprises two aspects: managing distress and changing external stress-evoking contexts to achieve adaptive outcomes, both aided by the evaluation of the need to regulate (cf. emotional awareness; Booth and Neill 2017; Ong and Thompson 2019; Skinner and Zimmer-Gembeck 2007; Ward et al. 2021). Two main types of coping have therefore been distinguished based on their targets: problem-focused and emotion-focused. Problem-focused coping addresses the sources of stress and is associated with better psychological outcomes as well as fewer behavioral problems (Compas et al. 1988; Hampel and Petermann 2006; Jamieson et al. 2018; Noret et al. 2018; Steinhausen and Metzke 2001). Emotion-focused coping, in contrast, regulates distressing emotions when provoking situations are seen as unlikely to be alterable, which tends to erode resilience (Horwitz et al. 2011; Hussong et al. 2021; Seiffge-Krenke 1993; Steinhausen and Metzke 2001). It has therefore been considered to be less effective for adjustment outcomes (Baker and Berenbaum 2007; Compas et al. 1988; Lazarus and Folkman 1984). Another potential strategy, social coping, involves support seeking to reduce negative emotions (Carver et al. 1989). Studies targeting early adolescents have reported positive associations between social coping and adjustment in response to problems. Seeking social help is influenced by self-efficacy and goals that individuals aim to achieve with regard to their social status, such as becoming popular (Rapee et al. 2019; Shin and Ryan 2012). According to teacher ratings, students with higher levels of self-efficacy, believing that their efforts will make a difference, employ more adaptive help-seeking behaviors and exhibit higher subsequent academic performance. Conversely, students who are concerned with appearing popular with others are less likely to engage in help-seeking. These reported associations all indicate that coping in its different forms is an important marker of individuals’ longer-term psychological outcomes (Compas et al. 1988, 1991; Wright et al. 2010). However, few studies have examined these processes in early adolescence, and since this is a critical period in terms of the developmental changes that occur, this requires attention.

In the current context, a key question concerns the sequence of the growth of emotion regulation and coping. Although research streams on ER and coping have remained relatively separate, both processes are activated under stressful situations, and possible connections have been suggested between them (Compas et al. 2014, 2017; Zalewski et al. 2011). Specifically, ER influences the selection of (i.e., forms a precursor to) coping strategies, and research indicates that deficits in both emotional awareness and regulation are negatively associated with adaptive coping (Messman-Moore and Ward 2014). Emotion dysregulation (poor control and expression of emotional responses) in the context of negative emotional experience has been considered as a characteristic that remains stable in accounting for negative affect. A greater extent of maladaptive ER tends to be associated with the decreased use of effective coping, which has been used to explain increased psychological disorders in models with clinical samples (Aldao et al. 2010; Hofmann et al. 2012; Thompson 2019).

Therefore, we theorize that there is a specific path linking the regulation of emotions and coping with the tendency to adopt negative ER strategies towards worry, modulating the selection of coping strategies (Gross 1998; Monteiro et al. 2014).

### 1.2. Emotional Awareness and Emotion Regulation

Emotional awareness is an attentional process that refers to the ability to identify, experience, and express emotions (Eastabrook et al. 2014). The literature on the adolescent development of awareness suggests that conscious awareness is a precondition of constructive management (Grove et al. 2017; Racine and Wildes 2013; Subic-Wrana et al. 2014; Zeman et al. 2010). More particularly, the ability to be aware of emotional experience is considered the initial stage of ER, as individuals need to recognize emotions internally before exercising regulation by employing appropriate strategies (Buckley and Saarni 2006; Penza-Clyve and Zeman 2002; Rueth et al. 2023). Emotional awareness as an antecedent stage of regulation has been measured through interoceptive awareness (Trevisan et al. 2019). Interoceptive awareness has been associated with the ability to attend to one’s physical and emotional cues, and impairments in awareness are linked to alexithymia, which is the failure to recognize emotions. Existing studies have also highlighted connections between emotional awareness, displays of worry (dysregulation), and the tendency to suppress emotions (inhibition), the latter two being considered as problematic regulation. Poor emotional awareness has been associated with a higher level of inhibition or dysregulation, suggesting confusion (Izard et al. 2011; Subic-Wrana et al. 2014), followed by blanket suppression or unregulated display. Poor awareness is also associated with maladaptive coping strategies (McLaughlin and Hatzenbuehler 2009; Suveg and Zeman 2004; Zeytinoglu et al. 2021). According to Penza-Clyve and Zeman (2002)’s study, preadolescents who reported a lack of emotional awareness were more likely to adopt maladaptive emotion regulation and coping in response to anger and sadness. Problem-focused coping involves actively addressing stressors, while emotion-focused coping tends to be a reactive response at a later stage of regulation. We therefore hypothesize that lower emotional awareness of worry will correlate to the greater use of emotion-focused coping (Landy et al. 2022). This link has not yet been investigated during the transition to adolescence. Given that emotional awareness and understanding develop along with cognitive and social skills, they are likely to vary across the transition period (Cummings et al. 2022). Older pupils with greater emotional awareness have been found to report fewer emotion regulation difficulties than young pupils (Riley et al. 2019). Therefore, age-related patterns regarding worry regulation need further investigation.

### 1.3. Cognitive Components in Emotion Regulation and Coping

Two major cognitive components have been recognized to maintain worry: cognitive self-consciousness and the negative belief that worry is uncontrollable (Wells and Cartwright-Hatton 2004). These two dimensions both pertain to metacognition, a higher-order process appraising one’s cognitions, and its link to stress coping (Forrest-Pressley and Waller 1984). According to Wells (2004, p. 167), metacognitive belief refers to the “cognitive process … involved in the regulation and appraisal of thinking itself”. It has also been conceptualized by Flavell (1976) as the perceived importance or consequence of controlling thoughts regarding cognition and emotions (Palmier-Claus et al. 2011). Previous findings have suggested that cognitive self-consciousness, the appraisal of thinking, may affect the controllability of worry beliefs (Palmier-Claus et al. 2013): paying increased attention to thoughts without clear strategies to manage them may result in harboring more negative beliefs that worries are uncontrollable (Wilson and Hughes 2011).

Much of the evidence in the literature derives from the self-regulatory executive function (S-REF) model of mental disorders among the clinical population (Wells and Matthews 1994, 1996). This model emphasizes cognitive self-consciousness’ effects on emotional and self-regulatory processing, thus directing subsequent controlled plans (Cook et al. 2015; Georghiades 2004; Sica et al. 2007; Wells 2000). Based on S-REF, an increased level of cognitive self-consciousness enables individuals to become more aware of their thoughts and to evaluate the relationship between their feelings and external environmental demands and make decisions about using more problem-focused coping strategies (Brown et al. 1986, as cited in Spada et al. 2008; Flavell 1976). In contrast, perceived uncontrollability is associated with decreased problem-focused coping and increased emotion-focused coping, as individuals have poor control over their thoughts and environments (Leslie-Miller et al. 2024).Within and beyond the S-REF model, appraisal has played a significant role in cognitive processing by evaluating the impacts and outcomes of stressful events (Lazarus and Folkman 1984). This process has been seen as shaping the occurrence and dynamics of both emotion regulation (ER) and coping with contextual demands, and serves as the foundation for metacognitive constructs (Cole et al. 2004; Compas et al. 2014; Wang and Saudino 2011). Lazarus’s (1991) model suggests that individuals make a primary appraisal to determine whether situations are stressful, followed by a secondary appraisal assessing whether they have sufficient resources to cope (Zalewski et al. 2011). Zalewski et al. (2011) used stress-evoking tasks to collect empirical evidence regarding the association between ER, appraisal, and coping strategies. They found that participants engaging in positive appraisal (i.e., viewing situations as involving less challenge and rejection compared to their resources) in the emotion-eliciting contexts reported lower levels of dysregulation. This resulted in higher levels of problem-solving and lower levels of emotion-focused coping. Hence, cognitive appraisals serve a predictive role in determining coping responses, with the frequent use of positive appraisal associated with positive adaptation to stress among preadolescents (Booth and Neill 2017; Dennis et al. 2009). Metacognitive beliefs built on this appraisal model suggest that higher cognitive self-consciousness and the stronger controllability of thoughts may help individuals adjust their responses via the greater use of problem-focused coping strategies. However, these processes have been studied less often among adolescents than in adults (Alhurani et al. 2018; Donovan et al. 2017; Ong and Thompson 2019; Yih et al. 2019).

### 1.4. Shifts in Emotion Regulation, Coping, and Cognitive Mediators Across Early Adolescence

The period between the ages of 11 and 14 years is typically defined as early adolescence or preadolescence, marked by substantial growth in cognitive skills and socioemotional capacity (Blum et al. 2014; Labouvie-Vief 2015; Santrock 2001). Growing introspection regarding emotions during this stage may influence individuals’ emotional awareness and perceptions of worry (Mancini et al. 2013). Several studies have reported that older early adolescents engage more frequently in problem-solving strategies, reflecting emerging cognitive maturity that supports different coping approaches (Blakemore and Choudhury 2006; Brown et al. 1986; Sanchis-Sanchis et al. 2020; Seiffge-Krenke 2000). Similarly, Muris et al. (1998) noticed a closer link between the low perceived controllability of worries and the use of maladaptive ER strategies among children aged 8 to 13 years versus children in later adolescence, potentially reflecting shifts in emotional awareness and cognitive understanding (Izard et al. 2011; Lane and Schwartz 1987). Increased maturity in cognition may also mean that individuals begin to have well-established metacognitive beliefs by the age of 13 (Cartwright-Hatton et al. 2004; Wilson and Hughes 2011).

At the same time, though, studies comparing samples with smaller age differences have so far found no evidence for developmental changes in coping strategies (Mullis and Chapman 2000; Stark et al. 1989). While children have demonstrated an enhanced capacity to choose effective strategies during middle childhood, age differences have not been found to affect the use of ER in early to middle adolescence, typically between the ages of 14 and 17 (Morris et al. 2011; Silk et al. 2003). However, this might be due to the inconsistency in the specific emotions being assessed. Given the heightened negative affect and emotion fluctuations reported during early adolescence, including a peak emergence of mental illness at the age of 14, exploring the patterns of worry-related processing during the transition to adolescence may provide a clearer picture for understanding future adaptation (Kessler et al. 2005; Kovacs et al. 2008; Somerville et al. 2010).

In the present study, we anticipated that different patterns of relationship among ER, metacognitive beliefs, and coping would be observed across younger (11 to 12 years of age) and older (13 to 15 years of age) early adolescents. Among younger pupils, it was expected that emotion-focused coping would be associated with uncontrollability beliefs and lower levels of cognitive self-consciousness. On the other hand, among older pupils, we expected that problem-focused coping would be associated with higher levels of emotional awareness and cognitive self-consciousness, along with greater controllability beliefs. Additionally, we hypothesized that greater emotional awareness would be associated with less problematic emotion regulation. Given the limited work on social coping, we had no specific expectations regarding where this would fit in the developmental sequence.

### 1.5. The Present Study

**Figure 1:**
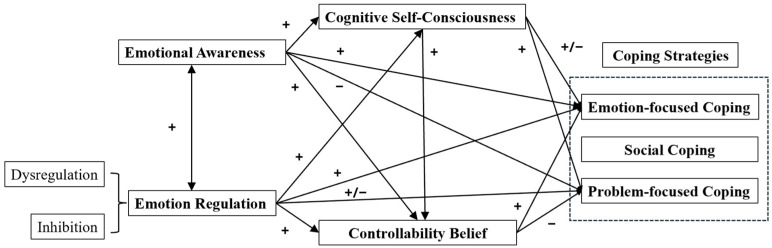
Proposed overarching model for emotion regulation, poor emotional awareness, metacognitive constructs, and coping strategies. Note: “+” and “−” signs indicate the hypothesized direction of associations. “+/−” indicates that the direction is expected to differ by age group or by dimension. Specifically, the relationship between cognitive self-consciousness and emotion-focused coping is positive in the younger group and negative in the older group. The relationship between emotion regulation and problem-focused coping depends on the strategies employed: dysregulation relates negatively to problem-focused coping, while inhibition relates positively to problem-focused coping.

The current study integrated emotion regulation, emotional awareness, metacognitive constructs, and coping into an overarching path model to examine how early adolescents respond to worry-eliciting situations (Figure 1). The model is framed around the formation of a negative mindset and how poor emotional awareness relates bidirectionally to two problematic ER strategies, inhibition, and dysregulation. These difficulties were expected to influence the two metacognitive constructs, cognitive self-consciousness and controllability of worries, which in turn shape coping strategies. We focused on the role of problematic regulation and negative controllability beliefs as potential risk factors for effective coping, i.e., the extent to which students view thoughts about emotions in certain situations as having unfavorable consequences and find these hard to handle. To clarify the effect of age differences on early adolescents’ mindsets in understanding worry, we also investigated the effect of age on the path between emotional awareness and ER in selecting adaptive (problem-focused) and maladaptive (emotion-focused) coping strategies.

Previous findings have mostly relied on questions assessing individuals’ general emotional experiences, rather than specific scenarios. This has limited our understanding of how coping might vary depending on the stressor. The present study therefore introduced five scenarios to identify potential changes in patterns of responding across contexts, namely school performance, peer relationships, physical health, family relationships, and appearance. These themes were based on seventh and eighth graders’ most common themes of worry-related problems introduced in a meta-analysis completed by Owczarek et al. (2020). This approach allowed us to examine whether ER and coping responses remain consistent or vary across different contexts. We addressed two key research objectives:

Objective 1: To investigate whether maladaptive ER strategies predict coping outcomes via reduced emotional awareness and negative metacognitive beliefs.

We hypothesized that dysregulation and inhibition would be associated with poor emotional awareness, which would in turn predict the decreased use of problem-focused coping and increased emotion-focused coping. We anticipated that these effects would operate both directly and indirectly through low cognitive self-consciousness and controllability beliefs. Due to limited evidence, we made no specific predictions for social coping in relation to metacognitive beliefs.

Objective 2: To investigate whether age moderates the relationship between metacognitive beliefs and coping.

We hypothesized that younger adolescents are more inclined toward emotion-focused coping, whereas older adolescents tend to adopt problem-focused coping, and that this difference is mediated by metacognitive constructs.

## 2. Method

### 2.1. Participants

A total of 354 sixth-grade pupils, with age variability within the grade, were recruited from two middle-sized elementary schools in southeast China. Both schools fully complied with the unified standards regarding students’ admissions required by the city board of education and entrance examinations, suggesting that they represent students with average academic performance and intelligence. Ethical approval was obtained from the Research Ethics Committee of University College London Institute of Education before data collection. Research details were sent to the headteachers to gain prior consent. Upon approval, an information sheet and an opt-in consent form were sent to students and parents, who were asked to complete and return it by a specific date. Data was only obtained from students who had gained parental consent.

In total, 15 (4.2%) out of the 354 responses were excluded from the analysis as these participants left at least one page of the survey instrument incomplete. Additionally, since data analysis was primarily based on age, one response with missing age was considered invalid and hence removed. The final valid sample consisted of 338 participants, of whom 48.4% were male and 50.9% were females (data for gender were missing for 1.5% of cases), with age ranging from 11 to 15 (*M_age_* = 12.6 years; *SD* = 0.7) (Table 1). Following the hypothesis that children from different age groups would present different ER patterns, two age groups were constructed, with the younger group consisting of participants aged 11 and 12 (*N* = 172; *M_age_* = 11.9 years; 52.9% females), while the rest were categorized into the older group (*N* = 166; *M_age_* = 13.2 years; 47.6% females).

### 2.2. Measures

The main survey contained both general and scenario-based questions. All questions were translated into Mandarin by the researcher and, as a check on consistency, reverse-translated and revised by three bilingual Chinese–English speakers.

### 2.3. General Questions

Emotion regulation. Participants’ levels of regulation towards worry were assessed by two factors, inhibition (tendency to suppress worry) and dysregulation (magnified worry display), as measured using items from the Children’s Worry Management Scale (CWMS; Zeman et al. 2010). Measures of inhibition and dysregulation consisted of four statements (e.g., “I hold my worried feelings in”) and three statements (e.g., “I cannot stop myself from acting really worried”), respectively. All statements were followed by a five-point Likert scale, ranging from 1 (Strongly disagree) to 5 (Strongly agree), with higher ratings indicating greater inhibition or dysregulation, apart from the first statement for inhibition (“I show my worried feelings”), which needed to be reverse-coded. The final scores of inhibitions and dysregulation were obtained separately by summing up all ratings within each factor. Total scores ranged from 4 to 20 and 3 to 15. Cronbach’s alpha was generated to check the measurements’ reliability, which were in the low but tolerable range (inhibition: *α* = 0.64; dysregulation: *α* = 0.57).

*Emotional awareness*. Individuals’ capacities for emotional awareness towards worry were measured by the poor awareness (lack of emotional awareness) subscale from the Emotion Expression Scale for Children (EESC; Penza-Clyve and Zeman 2002). The subscale consisted of eight items. An example item was “I have feelings that I can’t figure out”. Wordings were revised where necessary by replacing “upset” and “angry” with “worried”: “When I feel worried, I do not know how to talk about it”. Using a 5-point Likert scale (1 = Strongly disagree, 5 = Strongly agree), the total scores ranged from 8 to 40. Cronbach’s alpha values indicated that the measure of poor awareness (α = 0.71) showed acceptable internal consistency.

Metacognitive constructs. Participants’ metacognitive beliefs were assessed by two dimensions: controllability of worry (the levels of control participants feel over worry) and cognitive self-consciousness (the extent to which individuals focused on their own thoughts). Each dimension was measured using six questions, which were adapted from the “negative beliefs” and “cognitive self-consciousness” subscales of the Metacognition Questionnaire for Children Revised (MCQ-CR; White and Hudson 2016). A sample item from the controllability of worry dimension was “I can’t ignore my worrying thoughts”, and for the cognitive self-consciousness dimension “I think about my thoughts over and over”. The revised version of the scale was adapted from the original MCQ for adults (Cartwright-Hatton and Wells 1997). Items were rated on a five-point Likert scale (1 = Strongly disagree, 5 = Strongly agree), giving a minimum score of 6 and a maximum of 30 on each dimension. For cognitive self-consciousness, a higher score indicated a higher level of preoccupied attention to worrying thoughts. For the controllability dimension, a lower score represented a higher extent of control while a higher score suggested a greater lack of control over worrying thoughts. Cronbach’s alpha indicated acceptable internal consistency for both dimensions: controllability (α = 0.67), and cognitive self-consciousness (α = 0.82).

### 2.4. Scenario-Based Questions

To investigate individuals’ coping patterns within real-life scenarios, and whether this was consistent across various settings, five paragraphs were developed, with responses to each being made via ratings on three items adapted from the CWMS (Zeman et al. 2010): emotion-focused coping (suppression of negative emotions: “I keep myself from losing control of my worried feelings”), problem-focused coping (direct modification and elimination of sources of worry: “I try to calmly settle the problem when I feel worried”), and social coping (social support seeking: “I talk to someone until I feel better when I’m worried”). The scenarios were created based on five major worry themes commonly reported by adolescents in previous studies: school performance, peer relationships, family conflict, physical health, and appearance (Ang et al. 2007; D’Andrea 1994; Muris et al. 1998; Owczarek et al. 2020; see Supplementary Material). In particular, these themes were selected and adapted from peer-reviewed, validated work conducted in Asia (Ang et al. 2007), ensuring cultural relevance to the target participants. The wording of each paragraph was designed to reflect realistic and emotionally engaging situations familiar to students in the 11–15 age range, using first-person language. To ensure appropriateness, the content was reviewed by school-based gatekeepers with knowledge of students’ experience. For example, the vague theme of ‘personal concern’ was replaced by ‘appearance’, as this was found to be particularly salient for early adolescents. The following paragraph was an example that was created for the school performance theme:

“You just received the feedback from the last quiz. You are unhappy with your grade because it is below the class average. At the same time, your teacher starts to talk, ‘Everyone, please make sure you pay attention to what is taught in every lesson as there will be another quiz next week. Please be prepared’. Imagine: (1) You are worried about being not able to follow lessons (2) You start to get worried about not getting a better mark (3) You are worried about these quizzes will have impacts on your future.”

For each scenario, participants rated the likelihood of adopting each strategy with a five-point Likert scale (ranging from 1 = Very unlikely to 5 = Very likely). The total score for each coping scale was calculated as the sum of five responses across scenarios, with a minimum of 5 and a maximum of 25. The scenarios were tested through a pilot study with five sixth-grade students to ensure language appropriateness and applicability. Cronbach’s alpha was obtained for coping, emotion-focused (*α* = 0.86), problem-focused (*α* = 0.83), and social (*α* = 0.81), across five scenarios, suggesting high internal consistency in reported coping patterns across various contexts. The text of all five scenarios is provided in the Supplementary Materials.

### 2.5. Procedure

Following the school’s permission and ethical approval from University College London, the participants were recruited from nine sixth-grade homerooms by using cluster random sampling. Within each target school, four classes were randomly selected from the cohort, from which all students were recruited, subject to parental permission. The school headmasters were invited to check the language appropriateness of the survey, as the researcher was mindful that the scenario-based paragraphs might evoke different levels of concern. Additionally, homeroom teachers were provided with risk-mitigating information, such as contact information for the school’s mental health office.

For data collection, a paper-based version of the full questionnaire was distributed by each homeroom teacher, who also informed participants that their responses remained anonymous and confidential. Teachers were responsible for guiding the questionnaire completion and clarifying potential issues. It was also stated that the participation was voluntary, and participants could withdraw at any time without consequence. The questionnaire took 20–25 minutes to complete, and students were verbally debriefed by teachers regarding the research aim afterwards.

### 2.6. Statistical Analysis

The data were examined for missingness and outliers prior to performing inferential tests. Among the 338 responses, each measure contained fewer than 4 missing responses, indicated by Little’s MCAR test as being non-significant (*χ*^2^ (58) = 57.30, *p* = .50) (Little 1988). The missing values were replaced by an imputed value using full information maximum likelihood (FIML) estimates, extrapolating from the completed data. A post hoc power analysis was performed using G* Power 3.1.9.7. Based on the research conducted by Finkelstein-Fox et al. (2018) on the effect of mindfulness on coping flexibility, the anticipated effect size was small to medium when using Cohen’s (1988) criteria. With an *α* = 0.05, the power of younger and older groups to detect effects of this size was 99.31% and 99.16%, respectively, which was considered highly adequate.

We initially performed a repeated measures ANOVA to test whether different coping strategies were selected across the five contexts. We also used independent sample t-tests to examine differences between the two age groups for each of the study variables. Two separate correlation matrices were then developed to examine the relationships between variables that were the primary focus of our hypotheses. Correlation coefficients across the two age groups were transformed into z-scores for comparisons, and correlations showing statistically significant differences were noted. We then performed a moderation analysis with the PROCESS macro (Hayes 2013) to test the moderating effect of age on the relationship between cognitive self-consciousness and coping styles based on correlation results.

Finally, we conducted path analysis using SPSS (30.0) Amos to examine the tenability of the proposed model for students’ negative mindsets while contrasting the effect of age on the model in Figure 1. In line with the hypotheses, we tested separately for each group the indirect effects of the two ER strategies and poor awareness via the two metacognitive beliefs. We conducted 1000 bootstraps by using Maximum Likelihood Estimation. We modified the model within each group if our proposed one failed to demonstrate an acceptable fit. The model fit was assessed and interpreted as acceptable by reviewing the following criteria: comparative fit index (CFI) ≥ 0.90, goodness-of-fit index (GFI) ≥ 0.90, and root mean square error of approximation (RMSEA) ≤ 0.01, 0.05, and 0.08 for excellent, good, and mediocre fit, respectively (Bentler and Bonett 1980; MacCallum et al. 1996).

## 3. Results

### 3.1. Preliminary Analysis

Before testing hypotheses, we measured whether there were differences across the five coping contexts. Based on the repeated measures ANOVA, only within the use of social coping among the older group was there a significant difference across the five contexts, *F*(4, 656) = 3.18, *p* = .01. No statistically significant differences were found in the use of emotion-focused coping among the younger group, *F*(3.75, 637.04) = 0.17, *p* > .05, or older group, *F*(3.73, 612.13) = 0.79, *p* > .05. Similarly, no significant differences were reported in problem-focused coping for the younger group, *F*(3.62, 615.61) = 0.79, *p* > .05, or the older group, *F*(3.66, 600.32) = 0.98), *p* > .05.

Table 2 presents the means and standard deviations for each study variable, broken down by age group. As can be seen, although there are mean differences between groups, none of these were statistically significant (all *p* > .05). Younger participants reported higher inhibition and poorer awareness compared to older participants. Younger participants also had higher means for both metacognitive constructs, uncontrollability and self-consciousness. Regarding the coping variables, younger participants reported higher use of emotion-focused coping. While older participants had higher means for dysregulation, they also reported greater use of problem-focused coping and social coping than the younger group. Standard deviations were generally high in both age groups for all variables, underscoring that the extent of individual variability in responses outweighed age-related differences.

### 3.2. Correlations

Despite the lack of significant differences between age groups in the means of variables, we nonetheless observed changes in the relationships between emotion regulation, metacognitive beliefs, and coping between younger and older groups that indicated the substantial reconfiguration of worry-related processing. Within the younger group, inhibition was significantly associated with decreased dysregulation and less social coping, while dysregulation was significantly associated with elevated poor awareness and with the uncontrollability of worrying thoughts. Increased dysregulation was also associated with the reduced use of problem-focused coping and more social coping. Poor awareness was correlated significantly with both dimensions of metacognitive beliefs, uncontrollability and self-consciousness, and with decreased problem-focused coping. The perceived uncontrollability of worry among younger participants correlated significantly with more emotion-focused coping. Higher self-consciousness was also significantly linked with increased emotion-focused coping.

Among the older group, although inhibition and dysregulation remained inversely related, their associations with coping strategies shifted. Increased problem-focused coping was associated with increased inhibition, but was associated with decreased dysregulation. A significant negative correlation was found between lower levels of poor awareness and more problem-focused coping. As with the younger group, increased dysregulation correlated with more social coping. Of note, problem-focused coping was more prominent in the older group, showing significant correlations with both dimensions of metacognitive belief, uncontrollability and self-consciousness (Table 3).

Overall, the two age groups showed a significant discrepancy in the relationship between poor awareness and dysregulation, as well as between poor awareness and perceived uncontrollability, with both becoming sharper with age. There was also a significant difference in the association between cognitive self-consciousness and two of the three different coping strategies, as this shifted from predicting the use of emotion-focused coping towards problem-focused coping (Table 3). Based on the different group correlations, we conducted a moderation analysis to further test whether age interacted with metacognitive beliefs and coping. The results showed that age was a significant moderator of the positive association between cognitive self-consciousness and the use of problem-focused coping, *F*(1, 334) = 5.05, *p* = .001, Δ*R*^2^ = 0.01. Cognitive self-consciousness was a significant predictor of problem-focused coping in the older group, *b* = 0.35, *p* < .001, but this effect was not significant among the younger group, *p* > .05. Age was also a marginally significant moderator of the association between cognitive self-consciousness and emotion-focused coping, *F*(1, 334) = 2.69, *p* = .04, Δ*R*^2^ = 0.01, with a significant relationship only seen in the younger group, *b* = 0.28, *p* = .009, not the older group, *p* > .05.

### 3.3. Path Analysis

Following the conceptual map presented in the introduction, two path models were constructed for the younger and older groups with emotion-focused and problem-focused coping as outcomes, respectively. Social support seeking was not considered in the path model as it had no significant correlation with metacognitive constructs. Within each group, we investigated the relationship between poor awareness and problematic ER, and how these factors were linked with coping via the uncontrollability of worry and increased self-consciousness. Inhibition, dysregulation, and poor awareness were considered exogenous variables that had an effect on the uncontrollability of worry, cognitive self-consciousness, and emotion- vs. problem-focused coping strategies. We report direct and indirect effects of regulation strategies and metacognitive beliefs on outcome variables in Table 4 and Table 5.

### 3.4. Younger Group

**Figure 2:**
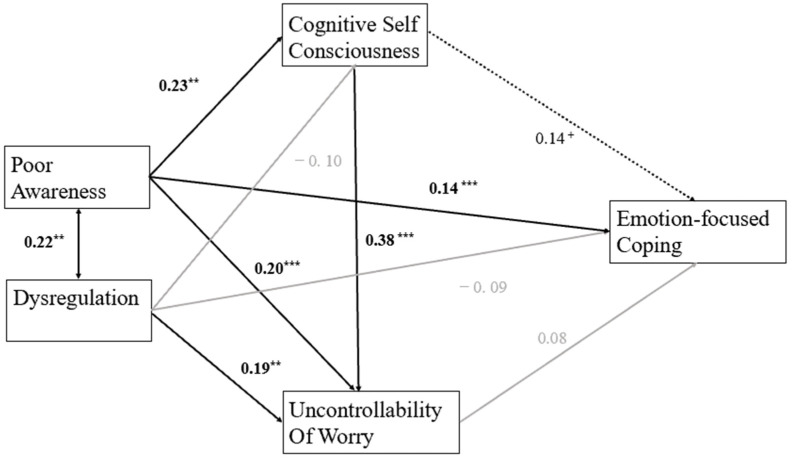
Path coefficients for relationships between dysregulation, poor awareness, uncontrollability of worry, cognitive self-consciousness, and emotion-focused coping among younger individuals. Note: The path between cognitive self-consciousness and emotion-focused coping is represented as a dashed line as the significance level was close to 0.05 (*^+^ p* = 0.05, ** *p* < 0.01, and *** *p* < 0.001).

The model assessed indirect effects of emotion dysregulation and poor awareness via cognitive self-consciousness and the uncontrollability of worry on coping. Inhibition was not included in the emotion-focused coping model (Figure 2) since it was only associated with dysregulation. The model had a good fit, χ^2^ = 1.492, GFI = 0.997, CFI = 0.994, RMSEA = 0.05. Poor awareness was significantly associated with dysregulation, *b* = 0.22, *p* = .005, as well as uncontrollability regarding worrying thoughts, *b* = 0.21, *p* < .001. Poor awareness was also significantly associated with a higher level of cognitive self-consciousness, *b* = 0.19, *p* = .01. The model also indicated a significant relationship between dysregulation and uncontrollability, *b* = 0.32, *p* = .01. Cognitive self-consciousness was related to a heightened level of uncontrollability, *b* = 0.34, *p* < .001, while displaying a positive association with emotion-focused coping, *b* = 0.18, *p* = .05.

A significant indirect effect was obtained from poor awareness to uncontrollability beliefs, *b* = 0.08, *p* < .05, and emotion-focused coping, *b* = 0.07, *p* < .05, via cognitive self-consciousness. A direct effect was found from poor awareness to uncontrollability beliefs, *b* = 0.28, *p* = .01. Cognitive self-consciousness also had a direct effect on uncontrollability beliefs, *b* = 0.36, *p* = .01. However, there was no significant direct effect from either cognitive self-consciousness or uncontrollability beliefs on emotion-focused coping.

When problem-focused coping and inhibition were added to the model, a poorer fit resulted, χ^2^ = 10.71, GFI = 0.98, CFI = 0.94, RMSEA = 0.11. There was no indirect effect found to be related to problem-focused coping. Therefore, the model outlined above was favored for the younger group.

### 3.5. Older Group

**Figure 3:**
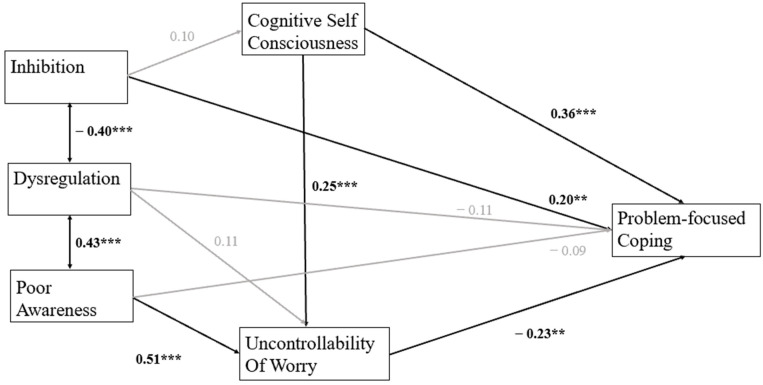
Standardized coefficients for relationships between inhibition, dysregulation, poor awareness, uncontrollability of worry, cognitive self-consciousness, and problem-focused coping among older participants. Note: ** *p* < 0.01, and *** *p* < 0.001.

Among older pupils, problem-focused coping was correlated with emotion regulation and the metacognitive constructs. Therefore, a path was added from inhibition to problem-focused coping via cognitive self-consciousness. A good fit was found for the resulting model, χ^2^ = 4.03, GFI = 0.992, CFI = 1.00, RMSEA = 0.00. Decreased dysregulation was significantly associated with increased inhibition, *b* = −0.41, *p* < .001, and increased inhibition was significantly associated with the increased use of problem-focused coping, *b* = 0.21, *p* = .01. While a significant relationship was found between dysregulation and poor awareness, neither of these two variables were significantly associated with problem-focused coping. It was noted that cognitive self-consciousness significantly contributed to both the heightened uncontrollability of worrying thoughts, *b* = 0.26, *p* < .001, and enhanced problem-focused coping, *b* = 0.36, *p* < .001. In contrast, there was a significant negative relationship between uncontrollability beliefs and increased problem-solving, *b* = −0.24, *p* = .005 (Figure 3).

Among older participants, significant indirect effects were obtained from poor awareness, *b* = −0.118, *p* < .001, and cognitive self-consciousness, *b* = −0.06, *p* < .001, to problem-focused coping via decreased uncontrollability of worrying thoughts. A significant direct effect from inhibition to problem-focused coping was also found, *b* = 0.21, *p* = .05, while dysregulation did not yield indirect or direct effects in this group (Table 5).

**Table 5:** Direct, indirect, and total effects for the older group.

	Dysregulation			Inhibition			Poor Awareness			Self-Consciousness			Uncontrollability		
	Direct	Indirect	Total	Direct	Indirect	Total	Direct	Indirect	Total	Direct	Indirect	Total	Direct	Indirect	Total
Uncontrollability	0.111	0.258 **	0.369 **	-	0.078	0.078	0.503 **	-	0.503 **	0.255 **	-	0.255 **	-	-	-
Problem coping	−0.104	−0.136 **	−0.24 **	0.21 *	−0.033 *	0.176 *	−0.096	−0.118 **	−0.214 **	0.36 **	−0.060 **	0.301 **	−0.235 **	-	−0.235 **

Note: * *p* < 0.05, ** *p* < 0.01.

## 4. Discussion

The current study investigated the association between emotion regulation (ER) and the selection of coping strategies among students between the ages of 11 to 15. Specifically, we tested direct and indirect effects of emotion regulation and awareness and two metacognitive constructs, cognitive self-consciousness and controllability beliefs, on coping strategies. Early adolescence is a critical developmental period characterized by changes in ER and the role of metacognitive constructs. This study aimed to better understand patterns of relationships among these constructs in the context of worry, as the existing evidence remains fragmented. To explore potential shifts in these patterns, participants were separated into two age groups. Although mean differences were not observed, high individual variability within groups and differential relationships between measures across groups suggest that early adolescence involves the reorganization of worry-related processing.

Based on significant correlations among variables, we developed contrasting path models for each age group, both with good fit. In the younger group (ages 11–12), dysregulation was negatively associated with poor emotional awareness, which was related to lower perceived controllability of worry. Lower perceived controllability was associated with greater cognitive self-consciousness. Poor emotional awareness was also directly associated with greater use of emotion-focused coping, and an indirect association was observed via cognitive self-consciousness. The older group (ages 13–15) fit into a model where lower dysregulation was related to greater inhibition, which was associated with the perceived controllability of worry. In turn, perceived controllability and cognitive self-consciousness were associated with greater problem-focused coping, while inhibition was also directly associated with greater problem-focused coping. An indirect association was also found between emotional awareness and problem-focused coping, via controllability beliefs of worry. These models suggest that the coping strategies used by two groups may reflect different patterns to managing worry-evoking situations, rather than a developmental difference. These findings extend past research by connecting emotion regulation and coping frameworks, suggesting that more adaptive coping was mediated by emotional awareness and the perceived controllability of emotions.

Emotional awareness is commonly accepted as the foundation for adaptive ER and was therefore treated as the starting point for responses (Rieffe et al. 2008). Our first hypothesis was that problematic emotion regulation strategies, specifically dysregulation and inhibition, would be associated with poor emotional awareness, which in turn would be linked to increased emotion-focused coping and decreased problem-focused coping. These associations were expected to be both direct and indirect, mediated by uncontrollability beliefs and lower cognitive self-consciousness. Our second hypothesis posited two different paths linking metacognitive beliefs to coping. For the younger group, lower uncontrollability of worry would predict the greater use of emotion-focused coping. For the older group, greater cognitive self-consciousness and higher perceived controllability of worry were expected to be linked, along with lower problematic emotion regulation, to greater problem-focused coping, which was considered to be more adaptive.

Consistent with our first hypothesis, individuals with poor emotional awareness were more likely to show a dysregulated response towards worry across both age groups. This supports previous findings suggesting a positive link between greater awareness and more adaptive regulatory strategies among early adolescents (Riley et al. 2019). Furthermore, the relationship between regulation and emotional awareness became stronger with age, which might indicate that adaptive emotion regulation during adolescence may increasingly rely on the development of the capacity to understand complex feelings (Eastabrook et al. 2014). Also, in line with the first hypothesis, we also found that emotion-focused coping among the younger age group was associated with poor awareness. This relationship between poor awareness and increased emotion-focused coping was partially mediated by cognitive self-consciousness, which also led to lower perceived controllability of worries. This suggested that there were two initial routes to managing emotions among early adolescents: one was driven by lack of awareness, resulting in unregulated displays; the other was based on some level of awareness but also resulted in lower perceived controllability of worry.

However, contrary to prior studies—and hypothesis one—inhibition did not show significant association with poor awareness. Instead, inhibition was, if anything—in the older age group—positively associated with adaptive problem-focused coping, suggesting that this may form part of a beneficial strategy. This aligns with cross-cultural studies indicating that suppression/inhibition may be perceived as adaptive in certain conditions, particularly in Asian cultures, where it is associated with effective emotion management when problems are manageable (Deng et al. 2017; Ong and Thompson 2019; Soto et al. 2011), so this may to some extent be a sample-specific effect. The negative relationship between dysregulation and inhibition also shifted across early adolescence, becoming increasingly negative with age. This pattern may reflect a tendency for inhibition to be used more deliberately as one alternative to poorly regulated displays, as adolescents gain greater inhibitory control over their emotional responses to match environmental demands. Inhibition may facilitate faster responses in contexts where downregulating emotion reactions are needed, such as during implicit association tasks (Deng et al. 2017).

Our findings indicated that social-focused coping only correlated with increased dysregulation, not inhibition. When worry was dysregulated, both younger and older participants were likely to seek social support, consistent with prior work suggesting that worry expression plays a part in interpersonal interactions (Rose 2002). Expressing worry provides chances for finding solutions, alerting, as well as co-rumination (Parkinson and Simons 2012). However, contrary to previous literature, social support seeking was not associated with either of the two metacognitive constructs, suggesting that it is a low-level strategy that could be maladaptive and driven by an abdication of responsibility for emotional responses. Clinical studies have similarly reported that help-seeking was linked to increased mental health symptoms, possibly due in part to a misalignment between help-seeking and the available resources in the immediate environment (Simione et al. 2021).

Consistent with our second hypothesis, we identified two different path models characterizing the relationships among key variables for younger and older early adolescents. In the younger age group’s model, emotion-focused coping appeared to stem from poor emotional awareness, rather than the perceived uncontrollability of worry. This may reflect an initial emotional response to only partly understood worry, rather than directly addressing challenges in situations. This relationship was partially mediated by increased cognitive self-consciousness. This suggests that younger pupils might have recognized the presence of their active thoughts but lacked a deeper understanding, given their limited cognitive resources during this transitional stage (Eastabrook et al. 2014; Laugesen et al. 2003). The link between self-consciousness and uncontrollability beliefs that was also noted may be explained by younger pupils’ insufficient self-efficacy to perceive emotional thoughts as controllable (Palmier-Claus et al. 2013; Tamir and Mauss 2011). For instance, Sweeny and Dooley (2017) suggested that self-focused thoughts are likely to promote rumination and repetitive thoughts, which are related to decreased cognitive control.

In contrast, as expected, the older group exhibited a shift toward problem-focused coping. This shift suggests a change in the organization of metacognitive and emotion processing, with cognitive self-consciousness becoming an independent predictor of problem-solving. This positive association between self-consciousness and problem-focused coping supports previous findings that advanced levels of thinking enhanced the capacity for adaptive adjustment, such as initiating problem-solving (Bailen et al. 2019; Palmier-Claus et al. 2013; Sica et al. 2007). However, cognitive self-consciousness also remained positively associated with the perceived uncontrollability of worry among younger participants, while perceived uncontrollability was negatively associated with problem-focused coping for older participants. This indicates that self-consciousness contained both maladaptive and adaptive components, supporting problem-solving in some contexts, but also relating to self-obsession and confusion in others (Rankin et al. 2004; Wells and Matthews 1994). This contradictory pattern of high cognitive self-consciousness but low emotion awareness may reflect individual differences in the use of metacognitive resources: for some adolescents, heightened self-focus may support regulation, while for others it may increase distress. Prior studies have linked balanced self-awareness with positive affects, whereas excessive self-focus may contribute to negative appraisals and internalizing symptoms (Higa et al. 2008).

Contrary to our expectations based on prior works (Endler et al. 2000; Palmier-Claus et al. 2013), the controllability of worry did not directly predict emotion-focused coping, and only directly predicted problem-focused coping. Considering that controllability beliefs are a key factor of emotion regulation (Ford and Gross 2019), they did not directly shape coping in response to worry-related stress. Instead, their indirect influence on emotion-focused coping appeared to go through cognitive self-consciousness. These findings suggest that cognitive self-consciousness may play a more immediate role in coping during early adolescence and that there are differences between emotion regulation and coping.

## 5. Limitations

This study has several limitations that should be considered. First, with the exception of social coping, individuals’ choice of coping strategies was highly consistent across the five scenarios used. This might suggest that the scenarios did not provide sufficiently varied contexts to elicit different coping responses and perceived controllability of situations (Compas et al. 1991; Fisher 1984, as cited in Wells and Matthews 1994). Nevertheless, the observed differentiation in the use of social coping responses suggests that participants did attend to scenario content to some extent. Additionally, the age-related variation in model structure and shifts in the predictors of coping indicate that coping responses were not entirely uniform. These findings may reflect the presence of underlying response ‘styles’—patterns of responding that persist across situations but still adapt to contextual demands. This possibility needs further exploration, perhaps tested by a wider group of participants in this age range.

Second, we did not collect information on individual variables such as academic performance or socioeconomic status (SES). SES has been reported to influence the associations between emotional experience and mental health across multiple developmental stages, including early adolescence (De France and Evans 2021). Moreover, academic performance and academic stress have previously been linked to disengaged coping strategies, highlighting their relevance to the present work (Arsenio and Loria 2014). These factors and their associations with coping and its underlying metacognitive beliefs need further investigation. Although gender was included as a covariate in our path models, it was not considered as a primary grouping variable, and therefore not subjected to detailed analysis. Additionally, our study sample was drawn from a limited number of schools, which may limit demographic diversity and the representativeness of findings. Future research should aim to recruit more diverse samples and consider gender as well as other demographic variables as potential moderators. Third, coping was assessed through scenario-based questions, while emotion regulation was assessed through general questionnaire items. It is possible that individual differences in emotion regulation were influenced by how worry was processed in each context, given that emotion regulation is context-dependent (Kuppens and Verduyn 2015). Future studies should consider alternative approaches to determine individuals’ reactions to negative scenarios, combining physiological measures and concurrent measures to assess responses to worry-eliciting tasks (Zalewski et al. 2011). Fourth, the study’s assessment of poor emotion regulation focused only on dysregulation and inhibition. Other regulatory difficulties, such as rumination or avoidance, may also play a role in shaping negative cognitive patterns and coping (Hong 2007; Li et al. 2006). Future work should explore a broader range of regulatory strategies to capture a more complete path. Finally, while internal reliability for all coping subscales was strong, the adapted scenario-based items need further validation. This could involve examining their test–retest reliability and validity through associations with other established constructs, such as perceived stress. Future research should test the generalizability of these measures across more diverse populations.

## 6. Implications and Conclusions

While not all early adolescents experience heightened emotional arousal or uncontrollable worry, for many, intensified self-consciousness and poor emotional awareness may elevate confusion, leading to unregulated behaviors (Eastabrook et al. 2014). Although we did not directly examine outcomes of these regulatory paths, the observed patterns indicated a need to support developing worry mindsets. From a practical perspective, early adolescents may benefit from opportunities to label and explore emotions collaboratively, strengthening both emotional awareness and regulation. Instruction should aim to validate stressful emotions while modeling flexible coping strategies. Educators and caregivers play a key role in creating environments that encourage pupils to reflect on their thoughts. When embedded within collective classroom settings, these skills may equip early adolescents to navigate complex emotional and social situations.

Overall, this work provides empirical evidence that coping during early adolescence is shaped by the interplay between emotion regulation and metacognitive processes. Among younger participants, emotion-focused coping was directly associated with poor emotional awareness, while cognitive self-consciousness and the perceived uncontrollability of worry appeared to reflect internal processing but did not independently predict coping. Second, problem-focused coping was associated with cognitive self-consciousness both directly and indirectly via reduced uncontrollability beliefs. These findings highlight a shift in the organization of worry-related processes regarding how self-reflective thinking contributes to adaptive coping, calling for further investigations of ER and coping shaping socioemotional adjustment outcomes in youth.

## Figures and Tables

**Table 1:** Demographic characteristics of students divided by group.

Group	Variable	Category	Frequency	Percentage
Younger group	Age	11	16	9.3
(*N* = 172)		12	156	90.7
	Gender	Male	78	45.3
		Female	91	52.9
		Missing	3	1.7
Older group	Age	13	138	83.1
(*N* = 166)		14	27	16.3
		15	1	0.6
	Gender	Male	86	51.8
		Female	79	47.6
		Missing	1	0.6

**Table 2:** Descriptive statistics and mean score comparisons between age groups for each of the study variables.

	Younger Group		Older Group		*t*(df)	*p*	95% CI	
	*M*	*SD*	*M*	*SD*			Lower	Upper
Inhibition	13.33	2.89	13.02	2.88	0.14	0.71	−0.27	0.98
Dysregulation	6.20	2.06	6.42	2.39	1.19	0.28	−0.71	0.24
Poor awareness	24.44	5.21	23.97	5.23	0.07	0.79	−0.76	1.48
Uncontrollability	18.30	4.07	17.53	4.42	0.88	0.35	−0.23	1.60
Self-consciousness	22.50	4.19	22.17	3.85	1.18	0.28	−0.55	1.18
Emotion-focused coping	15.86	4.81	15.36	4.82	0.02	0.88	−0.53	1.54
Problem-focused coping	18.33	4.14	18.42	3.72	0.59	0.44	−0.94	0.75
Social coping	16.41	4.44	16.46	4.58	0.68	0.41	−1.02	0.92

**Table 3:** Mean, SD, correlation, and coefficient differences among study items across the two age groups.

Variable	1	2	3	4	5	6	7	8
Younger Group								
1. Inhibition	-							
2. Dysregulation	−0.39 ***	-						
3. Poor awareness	−0.04	0.20 **	-					
4. Uncontrollability	−0.01	0.21 **	0.40 ***	-				
5. Self-consciousness	0.14	−0.02	0.20 ***	0.40 ***	-			
6. Emotion-focused coping	0.14	−0.05	0.04	0.16 *	0.20 **	-		
7. Problem-focused coping	0.14	−0.18 *	−0.20 **	−0.12	0.06	0.22 **	-	
8. Social coping	−0.26 ***	0.20 **	0.03	0.10	0.02	0.08	0.11	-
Older Group								
1. Inhibition	-							
2. Dysregulation	−0.41 ***	-						
3. Poor awareness	−0.05	0.42 ***	-					
4. Uncontrollability	−0.01	0.34 ***	0.56 ***	-				
5. Self-consciousness	0.10	0.05	0.10	0.31 ***	-			
6. Emotion-focused coping	0.12	0.00	0.11	0.06	−0.04	-		
7. Problem-focused coping	0.31 ***	−0.30 ***	−0.25 ***	−0.23 ***	0.30 ***	0.16 **	-	
8. Social coping	−0.11	0.20 **	−0.01	−0.01	0.02	0.12	0.11	-
Correlation Coefficient Comparison								
1. Inhibition	-							
2. Dysregulation	0.22	-						
3. Poor awareness	0.13	−2.38 *	-					
4. Uncontrollability	0.45	−1.31	−2.21 *	-				
5. Self-consciousness	0.30	0.88	1.07	1.06	-			
6. Emotion-focused coping	0.14	−0.68	−0.32	1.15	2.15 *	-		
7. Problem-focused coping	−1.48	1.27	0.39	1.08	−1.96 *	0.14	-	
8. Social coping	−1.41	0.02	0.36	0.97	0.04	−0.40	−0.03	-

* *p* < .05, ** *p* < .01, and *** *p* < .001.

**Table 4:** Direct, indirect, and total effects for the younger group.

	Dysregulation			Poor Awareness			Self-Consciousness			Uncontrollability		
	Direct	Indirect	Total	Direct	Indirect	Total	Direct	Indirect	Total	Direct	Indirect	Total
Self-consciousness	−0.102	-	−0.102	0.234 *	-	0.234 *	-	-	-	-	-	-
Uncontrollability	0.166 *	−0.036	0.13	0.280 **	0.083 *	0.363 **	0.356 **	-	0.356 **	-	-	-
Emotion coping	-	−0.005	−0.005	0.005	0.07 *	0.075	0.160	0.032	0.193 *	0.090	-	−0.09

Note: * *p* < 0.05, ** *p* < 0.01.

## Data Availability

The original data presented in the study are openly available in OSF at (https://osf.io/h7nes/, accessed on 2 June 2025).

## Supplementary Materials

The following supporting information can be downloaded at: https://www.mdpi.com/article/10.3390/jintelligence13080090/s1, Scenario-based questions.
