# Supplementary material for: Pathways of Worry During the Transition to Adolescence: An Exploration of Students’ Emotion Regulation, Metacognitive Beliefs and Coping
Source: J Intell. 2025 Jul 22;13(8):90. doi: 10.3390/jintelligence13080090 (PMC12387488; doi:10.3390/jintelligence13080090)
Supplement: Supplementary file 1 [file jintelligence-13-00090-s001.zip › jintelligence-3709747-supplementary.pdf]

Supplementary material:

Scenario-based questions

1. School performance	You just received the feedback from the last quiz. You are unhappy with your grade because it is below the class average. At the same time, your teacher starts to talk, "Everyone, please make sure you pay attention to what is taught in every lesson as there will be another quiz next week. Please be prepared".<br>Imagine:<br>1)You are worried about being not able to follow lessons<br>2)You start to get worried about not getting a better mark<br>3)You are worried about these quizzes will have impacts on your future
2. Peer relationships	As the new term begins, your class is holding a student election and you are thinking of running for a role in the election. Class voting is happening this afternoon and according to the rules, the position will go to those who get the highest vote. You understand that when a particular student gets elected for the role, it is because they are perceived as being more capable or well respected by their peers.<br>Imagine:<br>1)You are worried about how others perceived you<br>2)you are worried about whether your friends respect you<br>3)you are worried about whether you are bonding with classmates after holiday breaks
3. Physical Health	You are feeling unwell before going to bed. However, you have never experienced such feeling before and it feels different from the normal cold or sore throat. You shared your feelings with your parents, and they told you to rest early and see how it goes next morning. Now, you are

	lying in bed and<br><br>Imagine:<br><br>1)you are worried about whether you will get worse<br>2)You are worried about whether it is a chronic issue, or it will get better
4.Appearance	You finished up the last sip of water in your water bottle just before the class was dismissed. Shortly after the bell rang, you grabbed your bottle and planned to fill it up before the next class begins. Now you are walking down the aisle, and you feel like someone is staring at you.<br><br>Imagine:<br><br>1)You are worried about your look<br>2)You think about whether you look weird<br>3)You are worried about getting teased.
5. Family relationship	Your parents had a quarrel over some family issues this morning when you woke up. They seemed to switch the topic deliberately to keep you from noticing. Now you are not sure what happened and whether you should talk to them about this.<br><br>Imagine:<br><br>1)You are worried about their relationships<br>2)You are guessing why they argued and worried about whether someone had made mistakes.<br>3)You are worried about the arguments accelerated.
